# Integrated microfluidic systems for fluorescence monitoring rapid kinetic reactions in bioanalysis

**DOI:** 10.1007/s00604-023-05786-z

**Published:** 2023-05-11

**Authors:** Ángela Écija-Arenas, Antonio Zafra-Poyato, Juan Manuel Fernández-Romero

**Affiliations:** Departamento de Química Analítica, Instituto Químico para la Energía y el Medioambiente (IQUEMA), Universidad de Córdoba, Campus de Rabanales, Edificio Marie Curie, Córdoba, E-14071 España

**Keywords:** Alkaline phosphatase, Integrated microfluidic fluorimetric biosensor, 3D printed alignment device, Stopped-flow microfluidic, Enzyme-magnetic nanoparticles, Sulfonamides

## Abstract

A stopped-flow microfluidic fluorimetric biosensor to monitor alkaline phosphatase (ALP) activity and evaluate the potential inhibitors has been developed, integrating a magnetically retained enzyme microreactor (MREµR) in the reaction/detection zone of the microfluidic chip. The integration supposed the alignment of the MREµR at the sample compartment of a conventional spectrofluorometer using a 3D-printed device. The analytical signal is based on the fluorescence decrease in the signal obtained in the dephosphorylation reaction of the substrate 4-methylumbelliferone phosphate (4-MUP) by the retained ALP-MNPs in an alkaline medium caused by sulfonamides. The excitation and emission wavelengths to monitor the reaction were 363 and 444 nm, respectively. Three sulfonamides, acetazolamide, furosemide, and sulfasalazine, have been used as model analytes. The front-face operating mode of the spectrofluorometer was used to acquire the instrumental signals. The influence of the rotation angle of the microfluidic device on the efficiency of the signal collection has also been studied, obtaining the signals with greater intensity at 75° from the excitation beam. The dynamic range of the calibration graph was 16.81–1111.22 µg mL^−1^, expressed as sulfonamide concentration, with a limit of detection of 5.04 µg mL^−1^ (*R*^2^ = 0.9989, *n* = 10, *r* = 3) for acetazolamide. The method was applied to determine sulfonamide residues in tap water and milk samples, with 88.9–98.7% recovery values. The results have been compared with those obtained using a commercial device connected to the spectrofluorometer, getting faster reaction kinetics.

**Graphical abstract:**

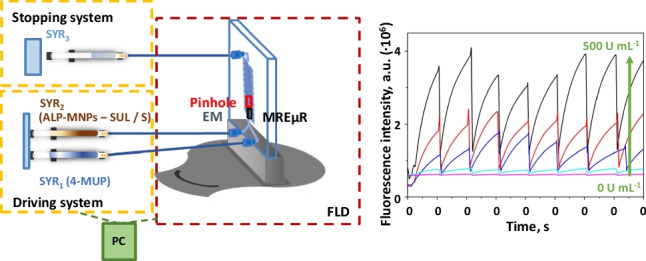

**Supplementary Information:**

The online version contains supplementary material available at 10.1007/s00604-023-05786-z.

## Introduction

In the last 4 decades, micrometer-scale systems have received significant attention from the scientific community, being a valuable tool in various research, development, and innovation areas, such as the synthesis of new materials, the development of automatic (bio)chemical analysis systems, or in control of chemical/biological processes, among others [1, 2]. Therefore, the different possibilities these automatic systems provide make them suitable for monitoring enzyme kinetics [3, 4]. An example of rapid response kinetic methodologies is the development of stopped-flow systems (SFS) [5, 6]. These systems facilitate the rapid mixing of the ingredients of the analytical reaction and their kinetic monitoring when the flow stops in the observation cell. These systems allowed the study of enzymatic reactions [7].

However, one limitation of these systems is conditioned by the sample and/or reagent volumes needed in order of milliliters. The use of miniaturized systems has been addressed to solve this problem. An integrated system between the SFS and a microfluidic system (µSFS) was developed. Microfluidic systems can integrate different stages of the analytical process on a single chip. One of the main disadvantages of microfluidic systems is the complex mixture due to the laminar regime produced in them.

Nevertheless, the strong impact of rapid drive and mixing reaction ingredients minimizes the initial effect of a system operating in laminar flow mode and facilitates the rapid mixing due to the turbulence created. In this way, it is easier to quickly reach the physical balance of diffusion of the reactants and the kinetic development of the enzymatic reaction. On the other hand, the advantages of working in a microscale device are lower sample and reagent consumption, shorter analysis time, higher sampling frequency, and portability [8, 9].

Monitoring the analytical signals in the proposed µSFS is based on observing the biocatalytic reaction by fluorimetry. When integrating the reaction/detection zone of a microfluidic system with a conventional spectrofluorometer, two options for focusing the optical beam on the microfluidic device can be considered, by coupling off-chip elements used in the macroscale, such as optical fiber or 3D-displacement devices, or incorporating them as miniaturized elements directly on-chip. These two options should consider studying many variables, integrating voluminous and/or expensive equipment, and a complex development process [10, 11]. An intermediate option supposed the integration of the microfluidic device in the conventional detector using elements that facilitated the focusing of the microchannel where the reaction/detection occurred in the optical detector pathway. This option involves incorporating an alignment device manufactured using 3D printing technology to integrate the microfluidic device [12].

The developed microfluidic system has been applied to perform kinetic-enzymatic studies with the enzyme alkaline phosphatase (ALP), in which the minimization in the consumption of the enzyme was intended to reduce the cost, using the immobilization on magnetic nanoparticles (MNPs) [13, 14]. Immobilization of the enzyme has additional advantages, such as resistance to moderate pH, temperature, or ionic strength changes and the possibility of reuse [15, 16]. The immobilized enzyme could be collected in the reaction/detection zone of the µSFS to obtain a magnetically retained enzymatic microreactor (MREµR) [17].

The bioanalytical reaction supposed the use of a zinc metalloprotein, such as ALP, which performs its functions at alkaline pH, using the dephosphorylation reaction of the 4-methylumbellyferyl phosphate (4-MUP) substrate due to the luminescent properties of the reaction product [18]. The presence of different compounds can modify the activity of enzymes, and inhibitors are those substances responsible for reducing enzyme activity due to their presence in the reaction. Sulfonamide compounds were inhibition compounds of this enzyme, which caused a decrease in the analytical signal obtained in the dephosphorylation reaction [19]. This type of inhibitor showed characteristics of a mixed inhibition, having both competitive and noncompetitive characteristics, in which the inhibitor binds directly to the enzyme, retarding the enzyme–substrate complex formation rate, as well as to the enzyme directly to prevent the breakdown of the complex.

Sulfonamides are widely used in human and veterinary medicine. However, the excessive use of these drugs can lead to problems in different humans and livestock—for example, the development of antibiotic resistance or harm to food-producing animals. Furthermore, sulfonamides are challenging to degrade and can persist in the environment for long periods, increasing their damage [20, 21]. Therefore, it is crucial to develop methods to determine these compounds in foodstuffs intended for human consumption. The developed µSFS has been applied to study the type of inhibition with the immobilized enzyme and to obtain a method to determine them in pharmaceutical and agri-food samples.

## Materials and methods

Materials, apparatus, instruments, and the synthesis of magnetic nanoparticles and covalent immobilization of the enzyme are provided in the Electronic Supplementary Material (ESM).

### Enzymatic and analytical reaction

The selected enzymatic reaction for the development of this work involves the dephosphorylation of the 4-MUP substrate, where the ALP enzyme acted as a catalyzer in the presence of magnesium and zinc ions. The final product of the reaction is 4-methylumbelliferone (4-MU) and inorganic phosphate. The final product, 4-MU, presented fluorescence, emitting radiation at 444 nm when excited with an excitation beam of 363 nm. When sulfonamide compounds were involved in the reaction, a decrease in the fluorescence signal was observed. This signal change was because they were enzyme inhibitors, and the dephosphorylating reaction did not occur in the presence of these compounds. The signal decrease is directly related to the concentration of sulfonamides in samples.

### Stopped-flow microfluidic system

**Fig. 1:**
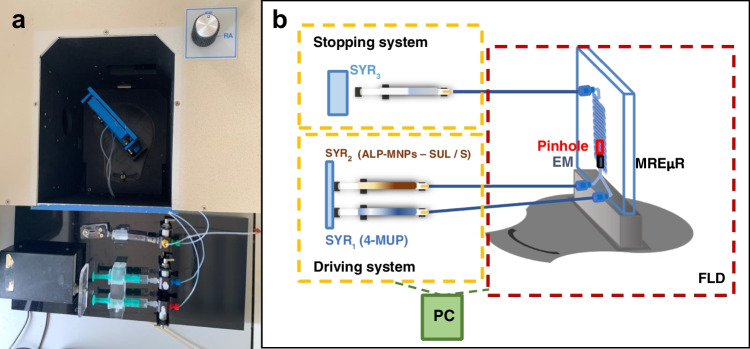
Configuration of the microfluidic stopped-flow system (µSFS) and signals acquired. **a** Image of the system integrated with the spectrofluorometer. **b** Scheme of the elements that make up the system. SYR_1_ and SYR_2_ denote driving syringes; SYR_3_, stopping syringe; ALP-MNPs, alkaline phosphatase immobilized on magnetic nanoparticles; SUL, sulfonamide compounds; S, sample; 4-MUP, 4-methylumbelliferyl phosphate; EM, electromagnet device; MREµR, magnetically retained enzymatic microreactor; FLD, fluorimetric detector; PC, personal computer

An adaptation of a stopped-flow module is proposed to be used with miniaturized components and obtain the µSFS. An image and a scheme of integrating the stopped-flow microfluidic device with the detector instrument are shown in Fig. 1 (Fig. 1a and b). It was composed of the driving system, the stopping system, and the microfluidic platform, including the chipholder with the microchip, which acts as the mixing chamber, the electromagnet, and the optical system. All parts were connected by poly(tetrafluoroethylene) (PTFE) tubes (i.d. 0.25 mm). The driving system consisted of two 2 mL glass syringes (SYR_1_ and SYR_2_) through which the reagent solutions, such as 4-MUP and ALP-MNPs first and sulfonamide solutions or samples later, were introduced in the mixing chamber. Each impulsion displaced a volume of 0.15 mL of a mixture from each syringe in the same proportion, which arrived at the mixing chamber in the microfluidic platform where the reaction started and the signal monitoring was carried out. The microfluidic platform was placed in the optical pathway of a conventional spectrofluorometer thanks to a 3D printed alignment device, as performed in previous research, rotated 75° from the excitation beam [12, 22]. The observation zone consisted of a pinhole located in the merging zone of the microfluidic chip, behind which an electromagnet created the MREµR. Once the mixture left the microreactor, it arrived at the stopping system, constituted by a 1 mL glass syringe (SYR_3_), and the flow was stopped, producing the start of the acquisition of the signal-time recording for approximately 50 s. All microfluidic measurements were carried out using the *front-face* acquisition mode of the spectrofluorometer, where the emission beam was recorded at a 22.5° angle from the excitation beam.

**Fig. 2:**
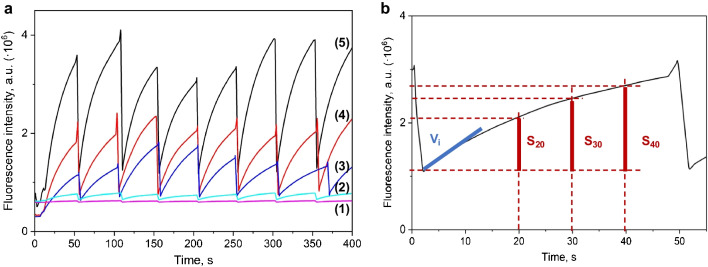
**a** An example of a series of eight injections at different increasing ALP activity, (1) 0, (2) 0.05, (3) 0.125, (4) 0.25 and (5) 0.5 U mL^−1^ **b** Instrumental signals acquired with the stopped-flow microfluidic system of reaction rate (v_i_) and signals at three times from the injection (s_20_, s_30,_ and s_40_)

The developed microfluidic system allows the retentions and preconcentration of ALP-MNPs in the reaction zone using an electromagnet, minimizing the reaction bolus and saving enzyme consumption. The experimental development required two steps. The first step was introducing 0.3 mL of a suspension containing ALP-MNPs in the working buffer with a 1:5 dilution factor to be magnetically retained in the microreactor and form the MREµR. This step was needed once for all measurements since the enzyme was reused. SYR_2_ introduced ALP-MNPs, while the working buffer was introduced through SYR_1_. The second step involved developing the analytical reaction between the ALP, 4-MUP, and sulfonamide compounds. SYR_1_ contained the 4-MUP solution in Tris–HCl buffer (10 mmol L^−1^, pH 9.8), while SYR_2_ was used to introduce sulfonamide solutions or samples in this second step. When the reagents for the analytical reaction entered the system, the flow was stopped automatically by the third syringe when a total volume of 150 µL of mixing both syringes in the same proportion was introduced in the system, and the kinetic tracking of the enzymatic reaction was achieved during 50 s. A series of measurements of eight successive impulses, as shown in Fig. 2a, were carried out, acquiring a series of eight repetitions in 400 s.

The efficiency of the designed system was proved by comparing results obtained with a conventional stopped-flow system. For this purpose, the first syringe introduced the reaction substrate at 0.2 mmol L^−1^. The second one contained enzyme solutions with different activities (0–0.5 U mL^−1^) in the case of the ALP activity study or a solution with an enzyme activity of 0.5 U mL^−1^ together with the inhibitor compound at different concentrations (0–5 mmol L^−1^) in case of the sulfonamide detection.

Once the instrumental signal was acquired, a systematic signal treatment procedure was performed to obtain two types of signals, as shown in Fig. 2b, the kinetic parameter of reaction rate (v_i_) and the net signals obtained at a fixed time (s_20_, s_30_ and s_40_), as shown. The reaction rate parameter was obtained as the slope of the first five seconds signal, fixed time signals were obtained by the difference between the signal at the studied time and the beginning of the kinetic curve, and the net signals were obtained as the difference between the absence (I_0_) and the presence (I) of sulfonamides. In addition to determining the inhibitors in different samples, a study of the type of inhibition has been carried out using the double reciprocal representation established by Lineweaver–Burk.

### Sample analysis

The method was applied to determine three different sulfonamide compounds as models of ALP inhibitors, such as furosemide and acetazolamide as non-antibiotic sulfonamides and sulfasalazine as an antibiotic sulfonamide. These compounds were analyzed in different natures, such as pharmaceutical or liquid samples, such as tap water, well water, and whole milk. Pharmaceutical formulations were processed, diluted in Tris–HCl buffer, and filtered using a nylon syringe filter with a pore size of 0.45 µm to separate insoluble components. The water samples were also filtered using this filter. Sample pretreatment for the milk samples obtained from supermarkets was done as follows [23]. First, 20 mL of the sample was mixed with 20 mL of a 20% trichloroacetic acid solution and stirred for 40 min. After this, the solution was centrifugated at 4 °C for 15 min at 11,830 × g, and the supernatants were collected and filtered through a 0.2 µm membrane, followed by dilution fivefold with Tris–HCl buffer.

A recovery study was performed due to the lack of signal response above the LOQ obtained in some samples, using the standard addition method by adding two different amounts of each drug to each sample before carrying out the sample pretreatment. All measurements were carried out in triplicate.

## Results and discussion

### Characterization of ALP-MNPs

The characterization of the synthesized materials is presented in the ESM.

### Study of the experimental variables of the method

All the variables that could affect the hydrodynamic of the microfluidic system, the reaction/detection zone, and the chemical variables influencing the process should be studied to obtain the kinetic-enzymatic parameters affecting the ALP-MNPs complex and show the effectiveness of the µSFS designed. The study has been carried out using the univariate method, taking as a reference analytical signal the net signal at 40 s (s_40_), at two concentration levels of the substrate 4-MUP (0.01 and 0.2 mmol L^−1^) and two enzyme activities (0.125 and 0.5 U mL^−1^). Each test was repeated in triplicate. The temperature of the laboratory has been controlled to develop all the research at the same temperature (25 °C). Table 1 shows all the variables studied, with the intervals under study, and the optimal values obtained. Furthermore, the detailed study of variables is described in the ESM.

**Table 1:** Study of the variables involved in the stopped-flow microfluidic system

Type of variable	Variable	Range studied	Optimal value
Instrumental	λ_excitation_, nm	300–700	363
	λ_emission_, nm	400–700	444
	Excitation/emission slit, nm	1–10	2/2
	PMT gain, V	200–950	950
Microfluidic	Rotation angle of the system, º	30–90	75
	Pinhole diameter, µm	-	Approx. 250
	V_reactor_, µL	6–13	6
Physical	Temperature, °C	-	25
Hydrodynamic	V_injection_, mL	0.05–0.5	0.1
	Time between injections, s	1–500	50
Chemical	pH	7–11	9.8
	[Tris–HCl], mmol L^−1^	5–100	10
	[4-MUP], µmol L^−1^	5–1000	10 and 200
	ALP-MNPs dilution	1–20	5

### Study of the kinetic parameters of the ALP-MNPs retained in µSFS

The kinetic parameters of the enzymatic reaction were studied to compare the results obtained from the developed µSFS with the results from a conventional SFS. The detailed comparison is shown in the ESM.

### Study of the enzyme inhibition in the microfluidic system

Its application to the evaluation of enzyme inhibition models has been proposed to demonstrate the usefulness of the integrated µSFS. The double reciprocal representation method of Lineweaver–Burk has also been established to explore the inhibition response. For this, furosemide as a model of sulfonamide compound has been studied as a potential inhibitor at two different concentrations (1 and 5 mmol L^−1^). The treatment of these data allows knowing the inhibition model and calculating the apparent inhibition constants (K_I_), estimated for each concentration value. It is expected to obtain a model of reversible inhibition with a mixed competitive and noncompetitive mechanism [24]. The data obtained from the representation at different inhibitor concentrations, including when no inhibitor is added, is presented in Table 2.

**Table 2:** Estimation of the inhibitor kinetic parameters

[Furosemide], mmol L^−1^	Double reciprocal adjustment Eq. ^(1)^			Kinetic parameters		K_I_, µmol L^−1^
	Slope ± SD (10^−5^)	Intercept ± SD (10^−4^)	R^2^	K_M_, µmol L^−1^	v_MAX_, µmol L^−1^ s^−1^	
0	4.94 ± 0.19	4.83 ± 0.22	0.995	0.11	2.11·10^3^	-
1	12.1 ± 0.24	6.96 ± 0.33	0.996	0.15	1.44·10^3^	6.64
5	2.15·10^2^ ± 19.9	5.09 ± 0.17	0.991	0.23	5.01·10^2^	7.76

^(1)^ where slope = 1/v_MAX_ and intercept = K_M_/v_MAX_

The results showed that the inhibition studied was of a mixed type, competitive and non-competitive. All the representations converge above the horizontal axis and at the left of the vertical axis, with different intercepts and increasing the slope with the inhibitor concentration. This fact led to increasing K_M_ and decreasing v_MAX_ whit the inhibitor concentration. The inhibitor constant calculated (K_I_) was close to 7.2 µmol L^−1^, similar to those obtained in the literature [24].

### Analytical features of the method for sulfonamides determination

The methodology has been applied to determine a family compound, sulfonamides, with the ability to inhibit the activity of the enzyme ALP from demonstrating the usefulness of µSFS with the enzyme magnetically retained (ALP-MNPs). All the variables affecting the system shown above have been fixed to define the analytical characteristics of the determination method. The kinetics have been monitored by adding different concentrations of different sulfonamides, such as furosemide, acetazolamide, and sulfasalazine. Each calibration curve was obtained using ten points and making each point triplicate. The initial rate obtained by subtracting the signal from the reaction without inhibitor to the signal has been represented from the corresponding sulfonamide concentration. Table 3 shows the analytical characteristics of the proposed method showing the parameters of the equation defining the methodological calibration curve, the LOD, and the dynamic linear range, calculated according to IUPAC recommendations [25], as well as the accuracy of the method calculated as a percentage of relative standard deviation in the minimum and maximum error zone of the curve. For that, 20 blank samples were measured to obtain the mean and standard deviation values to make parameter calculations.

**Table 3:** Analytical features of the µSFS method for sulfonamides determination

Analyte	Equation of the calibration curve (*n* = 10, *r* = 3) ^a^			LOD, µg mL^−1^	LOQ, µg mL^−1^	Linear range, µmol L^−1^	%RSD ^b^	
	a ± SD	b ± SD	R^2^				Min	Max
Acetazolamide	0.53 ± 4.5·10^−2^	3.5 ± 0.9	0.9995	5.04	16.81	16.81–1.11·10^3^	2.3	6.1
Furosemide	0.54 ± 1.1·10^−3^	246.7 ± 1.4	0.9989	7.84	26.19	26.19–1.7·10^3^	1.4	6.6
Sulfasalazine	0.52 ± 1.2·10^−2^	26.5 ± 2.1	0.9987	12.11	40.36	40.36– 1.9·10^3^	3.2	6.3

^a^where *y* = ax + b, being y = average reaction rate, µg mL^−1^ s^−1^ and x = [sulfonamide], µg mL^−1^

^b^RSD values were obtained at two concentration levels, corresponding to the LOQ (maximum error) and half of the linear range (minimum error), respectively

Values obtained for LOD and LOQ are lower than the maximum residues limit (MRL) for these drugs in samples [26].

### Application of the µSFS method

The developed method has been applied to determine different sulfonamides, such as acetazolamide, furosemide, and sulfasalazine, in pharmaceutical, drinking water, and milk samples to quickly determine those drugs. Table 4 summarizes the results obtained for those drugs in different samples. No signal response above the LOQ was obtained to water and milk samples; thus, a recovery study was performed, using the standard addition method, by adding two different amounts of each drug, 100 and 500 µmol L^−1^, to each sample and subtracting the results obtained from similarly treated unspiked samples in case of pharmaceutical samples. These concentrations were chosen to be in the linear range of the calibration curve and inside the MRL of these drugs in milk [26]. All measurements were carried out in triplicate. As can be seen in Table 4, the recovery percentages obtained ranged from 88.7 to 99.5%.

**Table 4:** Application of the method

Sample	Type of sample	Acetazolamide			Furosemide			Sulfasalazine		
		Declared/found ± SD^a^	Recovery, %		Declared/found ± SD ^a^	Recovery, %		Declared/found ± SD ^a^	Recovery, %	
			22.22 µg mL^−1^	111.12 µg mL^−1^		33.07 µg mL^−1^	165.37 µg mL^−1^		39.84 µg mL^−1^	199.19 µg mL^−1^
1	Pharmaceutical sample	250/237.6 ± 12.1	99.5 ± 4.1	98.8 ± 4.3	40/38.4 ± 4.1	98.6 ± 3.6	99.4 ± 2.6	500/496.5 ± 12.3	97.6 ± 3.2	96.9 ± 4.2
2	Tap water	n.q.^b^	98.7 ± 2.5	96.1 ± 3.9	n.q. ^b^	97.1 ± 6.7	94.3 ± 2.5	n.q.^b^	97.2 ± 5.1	95.6 ± 3.7
3	Tap water	n.q.^b^	92.3 ± 4.7	95.5 ± 3.4	n.q. ^b^	96.3 ± 3.4	95.5 ± 5.2	n.q.^b^	93.1 ± 6.2	92.8 ± 4.6
4	Whole milk	n.q.^b^	89.9 ± 5.9	94.1 ± 5.5	n.q. ^b^	90.7 ± 5.1	92.2 ± 2.3	n.q.^b^	94.5 ± 8.8	93.7 ± 7.2
5	Whole milk	n.q.^b^	91.2 ± 3.2	93.1 ± 6.1	n.q. ^b^	88.9 ± 8.1	91.4 ± 5.7	n.q.^b^	89.9 ± 5.9	88.7 ± 6.7

^a^Units: mg per tablet

^b^Analyte concentration lower than LOQ

## Conclusions

In this research, an integrated rapid mixing and the stopped-flow system have been developed based on dynamic systems at the microfluidic scale (µSFS), whose mixing chamber is coupled in the sample compartment of a conventional spectrofluorometer using an alignment device manufactured using 3D printing technology. A magnetically retained enzymatic microreactor (MREµR) has been incorporated into the reaction/detection zone of the system, which includes the ALP enzyme immobilized in magnetic nanoparticles (ALP-MNPs). Kinetic parameters of the enzymatic reaction in the µSFS have been studied using the fluorimetric signal obtained in the dephosphorylation of 4-MUP by comparison with a conventional SFS and the enzyme in solution. The integrated μSFS system has been applied to the fluorimetric determination of sulfonamides in pharmaceutical and agri-food samples as inhibitors of the ALP activity, and the kinetic parameters of the reaction with the ALP-MNPs enzyme for different concentrations have been characterized. There is a limitation in this method, which is the possible interference due to the working wavelength, to which different common species can give signals. However, this handicap could be solved for other applications using longer wavelengths. The development of integrated µSFS is a helpful tool for monitoring rapid kinetic reactions in bioanalysis.

## Supplementary Information

Below is the link to the electronic supplementary material.

Supplementary file 1 (DOCX 1.32 MB)

## Data Availability

The authors confirm that the data supporting the findings of this study are available within the article and its Supplementary Electronic Material.
