# Supplementary material for: Integrated microfluidic systems for fluorescence monitoring rapid kinetic reactions in bioanalysis
Source: Mikrochim Acta. 2023 May 11;190(6):209. doi: 10.1007/s00604-023-05786-z (PMC10175461; doi:10.1007/s00604-023-05786-z)
Supplement: Supplementary file 1 — (DOCX 1.32 MB) [file 604_2023_5786_MOESM1_ESM.docx]

**Electronic Supporting Material on the Microchimica Acta publication:**

**TITLE:** Integrated microfluidic systems for fluorescence monitoring rapid kinetic reactions in bioanalysis

**AUTHORS:** Ángela Écija-Arenas, Antonio Zafra-Poyato, Juan Manuel Fernández-Romero*

Departamento de Química Analítica, Instituto Universitario de Investigación en Química Fina y Nanoquímica (IUNAN), Universidad de Córdoba, Campus de Rabanales, “Marie Curie” Building Annex, E-14071 Córdoba, España

* Corresponding author email: qa1feroj@uco.es

Other authors email: Ángela Écija-Arenas, q92ecara@uco.es; Antonio Zafra-Poyato, q62zapoa@uco.es

ORCID: Juan Manuel Fernández-Romero, 0000-0001-8443-1358; Ángela Écija-Arenas, 0000-0003-3240-5769

**Materials and methods**

**Materials**

All chemicals used were of analytical grade. Iron (III) chloride (FeCl_3_), Iron (II) chloride (FeCl_2_), Zinc chloride (ZnCl_2_), and Magnesium chloride (MgCl_2_) were purchased from Panreac (Panreac Química S.L.U. Barcelona, Spain, https://www.itwreagents.com/iberia/es/home). 4-methylumbelliferyl phosphate (4-MUP) and N-(3-dimethylaminopropyl)-N′-ethylcarbodiimide (EDC) were purchased from Sigma-Aldrich (Merck Life Science S.L.U., Madrid, Spain, http://www.sigmaaldrich.com/ES/es). Sodium hydroxide, hydrochloric acid, disodium hydrogen phosphate, and Tris-HCl were purchased from Merck (Merck Group, Madrid, Spain, http://www.merckgroup.com/). Alkaline phosphatase enzyme (ALP) (EC 3.1.3.1) was purchased from Sigma-Aldrich and diluted in 50 % glycerol with Tris-HCl 5 mmol L^-1^, pH 7, MgCl_2_ 5 mmol L^-1^ and ZnCl_2_ 0.1 mmol L^-1^, with a specific activity of 6000 DEA U mg^-1^ (expressed in DEA units). Different enzyme dilutions were prepared in Tris-HCl buffer 10 mmol L^-1^ with cofactors MgCl_2_ 2.5 mmol L^-1^ y ZnCl_2_ 0.15 mmol L^-1^ at pH 9.8 (named working buffer).

All solutions were prepared in Tris-Cl 10 mmol L^-1^ buffer (pH 9.8), using deionized water purified with a Milli-Q system (Millipore S.A.S., Molsheim, France, www.millipore.com).

**Apparatus and instruments**

All luminescence signals were in a Horiba Scientific Fluorolog-3P spectrofluorimeter (Jobin-Yvon Technology, HoribaScientific, Francia, www.horiba.com/scientific). This instrument was equipped with two signal acquisition modes, obtaining the emission signal at a right angle and 22.5°, named *front-face* acquisition mode. This mode is recommended for special optical cuvettes or solid samples using microfluidic devices, considering them as solid structures due to the small optical pathway they present. All the information provided by the instrument was processed using the FluorEssence application software (Horiba Scientific) compatible with Origin 9.1.0 software (64-bit, OriginLab Co. 2013, Northampton, MA, USA). The Origin 9.1.0 software displayed the signal treatment and transformation, and the kinetic enzymatic parameters were obtained using a macro developed with Microsoft Excel 365 software.

The *lab-built* microfluidic stopped-flow device (µSFS) was manufactured with the same configuration as a conventional stopped-flow module, using 2 mL Luer-lock glass syringes. The system was connected using polytetrafluoroethylene (PTFE) connections and tubes with 250 µm inner diameter and perfluoro-elastomer (FFKM) splints. As a reactor, a microfluidic glass chip (FC_R150.332.2) with 12×24 mm dimensions and a 6 µL inner volume was inserted in a chipholder (FC_PRO-CH4515). The beam was reduced with a pinhole located in the merging zone after an electromagnet was also placed. All microfluidic materials were provided by Micronit (Micronit microfluidics, Enschede, Netherland, www.micronit.com). The microreactor was placed in the sample chamber of a spectrofluorimeter by a 3D-printed alignment device performed using polylactic acid (PLA-850) filament, which was modified using a biodegradable NatureWorks Ingeo 3D850-PLA. Furthermore, a commercial RX-2000 module (Rapid Kinetic System, United Kingdom, http://www.photophysics.com) has been used to compare the results obtained with the µSFS. This module was equipped with two 2.5 mL Kloehn glass syringes, Teflon connections, and a mixing zone integrated with the silicon flow cell, which has a 10 mm optical pathway and a variable injection volume between 0,1 and 1 mL.

Other apparatus, such as a conventional oven, an ultrasound bath, and an MPW-350R centrifuge (MPW Med. Instrument, Warsaw, Poland, www.mpw.pl) with a cooling chamber rotating, equipped with an angle rotor HSL-11199 (45º, 12×12×1.5 mL, max. speed = 18000 rpm, 24088×g RCF and r_min/max_ = 3.5/6.25) were used for the ALP-MNPs synthesis.

**Synthesis of magnetic nanoparticles and covalent immobilization of the enzyme**

The MNPs were obtained following the previously described co-precipitation method [1]. Once the MNPs were synthesized and cleaned, they were dried in an oven at 90°C until the weight remained constant. The ALP enzyme immobilization of the MNPs surface is a critical step in the performance of the biosensor. This step consisted of the covalent binding of the enzyme on the MNPs due to the crosslinking carbodiimide reaction [2]. First, 50-70 mg of dried MNPs were added to 1 mL of phosphate buffer (50 mmol L^-1^, pH 7.4), and the mixture was sonicated for 15 min after adding 0.5 mL of 0.02 g L^-1^ EDC solution in the phosphate buffer mentioned. Following the carbodiimide activation, 2.5 mL of 100 U mL^-1^ ALP solution was added, and the mixture was sonicated again for 30 min at 4°C. Finally, the suspension obtained with the immobilized enzyme was centrifuged at 3000 rpm for 20 min. The precipitates containing ALP-MNPs were washed with phosphate buffer (50 and 100 mmol L^-1^, pH 7.4) and centrifuged after each wash. Each supernatant was collected and saved in the fridge at 4°C until further use. The ALP-MNPs remained suspended in the working buffer until their use.

The efficiency of ALP immobilization on MNPs was evaluated using a batch method in which solutions with different ALP enzyme activities (0 – 2.5 U mL^-1^) prepared in the working buffer and supernatants collected during the synthesis was mixed with a fixed concentration of 4-MUP substrate (0.2 mmol L^-1^) prepared in Tris-HCl buffer (10 mmol L^-1^, pH 9.8). The increasing fluorescence intensity was monitored at the excitation and emission wavelength of the reaction product, and the initial rate values were calculated from the kinetic record. The stability of the immobilized enzyme was studied by monitoring the fluorescence signals achieved over time using different 4-MUP concentrations (0.01 – 0.5 mmol L^-1^).

**Results and discussion**

**Characterization of ALP-MNPs**

The synthesized materials were characterized with different techniques to obtain information about MNPs and ALP-MNPs. MNPs characterization was avoided because they have been widely characterized by various techniques and were discussed in previous research [3, 4]. The ALP immobilization efficiency on the MNPs was determined using the fluorimetric method described, obtaining a result of about 94.2 ± 0.5 %. This enzyme immobilization remains constant for at least two months, with lower than 10 % activity losses. On the other hand, the enzyme in the solution presented an activity loss of 60 % of its initial activity in the same period, highlighting the usefulness of enzyme immobilization.

**Study of the experimental variables of the method**

Figure S1 resumes the results obtained from the study of the most significant variables.


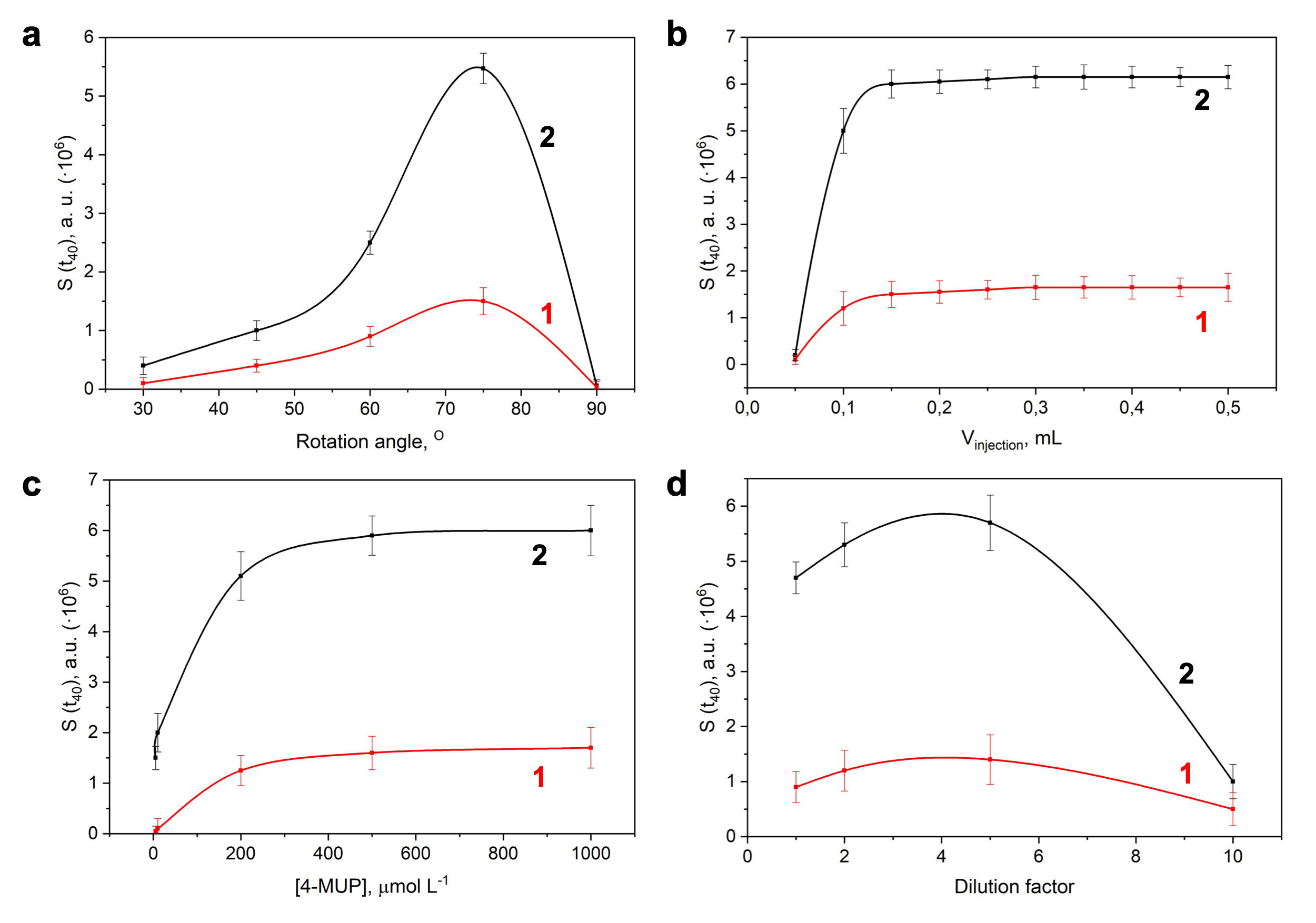


**Figure S1** Study of the variables affecting the stopped-flow microfluidic system: **a** Rotation angle at two enzyme activities, (1) 0.125 and (2) 0.5 U mL^-1^ **b** Injection volume at two enzyme activities, (1) 0.125 and (2) 0.5 U mL^-1^ **c** Concentration of the substrate 4-MUP at two enzyme activities, (1) 0.125 and (2) 0.5 U mL^-1^ and **d** Dilution factor of the ALP-MNPs complex at two 4-MUP concentrations, (1) 0.01 and (2) 0.2 mmol L^-1^

Among the variables of the microfluidic system shown in Table 2, the rotation angle of the microfluidic system is fundamental since both the excitation and emission beams must be focused on the microfluidic channel or the detector. The maximum signal is collected with a rotation angle of 75º from the excitation beam, as shown in Figure S1a. The pinhole is necessary to focus the excitation beam appropriately in a single channel and thus eliminate radiation scattering and interference, a fact that is achieved with a diameter of approx. 0.25 mm.

The hydrodynamic variables were studied since they affect the displacement on the microfluidic scale. The injected volume until the flow is stopped by arrival at the stop syringe (J_3_) has been studied, selecting a volume of 0.15 mL, as shown in Figure S1b. This volume has been chosen to guarantee the complete filling of the system, observing the most significant signal without interference from previous injections.

The study of chemical variables, such as the substrate 4-MUP concentration, has been carried out to obtain the maximum fluorescence signal in the characterization of the analytical parameters of the developed system. As shown in Figure S1c, high substrate concentrations led to the saturation of the enzymatic reaction, keeping the signal constant from 200 µmol L^-1^. Furthermore, to obtain the optimal MREµR, the amount of immobilized enzyme is essential to study. Figure S1d reflects no significant improvements when introducing solutions with a dilution greater than 1:5, retaining enough immobilized enzyme that interacts with the substrate without problems of signal dispersion, overpressure, and/or enzyme displacement.

**Study of the kinetic parameters of the ALP-MNPs retained in µSFS**

The kinetic parameters of the enzymatic reaction can be calculated from the development of the kinetic curves at different substrate concentrations and the calculation of the initial reaction rate, as indicated above. The enzyme has been immobilized inside the system to form the MREµR to perform this study in the µSFS. Measurements have been carried out varying the concentration of the substrate 4-MUP in the range of 0.5-1000 μmol L^-1^. The representation of doubles reciprocals, which corresponds to the Lineweaver-Burk linear fit, has been made to obtain the calculation of the K_M_ and v_MAX._ This method of linear adjustment has also been applied to calculate the kinetic parameters of the enzyme in solution using conventional SFS and thus obtain a comparison between both systems. Table S1 shows the results obtained from the linear representation and calculation of kinetic parameters (v_MAX_ and K_M_) for each design.

| **Table S1** Estimation of the kinetic parameters of the ALP-MNPs | | | | | |
| --- | --- | --- | --- | --- | --- |
| **Method** | **Double reciprocal adjustment equation ^(1)^** | | | **Kinetic parameters** | |
|  | **Slope** | **Intercept** | **R^2^** | **K_M_, µmol L^-1^** | **v_MAX_, µmol L^-1^ s^-1^** |
| **µSFS** | 4.94·10^-5^ (1.92 ± ·10^-6^) | 4.83·10^-4^ (2.22 ± ·10^-5^) | 0.995 | 0.11 | 2.11·10^3^ |
| **SFS** | 1.22·10^-2^ (± 2.04·10^-3^) | 1.09·10^-5^ (± 3.04·10^-6^) | 0.997 | 1.12·10^3^ | 9.19·10^4^ |
| ^(1)^ where slope = 1/v_MAX_ and intercept = K_M_/v_MAX_ | | | | | |

For both cases, the representation fits a straight line. However, the kinetic parameters do not have the same order of magnitude, justifying this in the difference in the optical step of both methods. In the conventional system, the optical passage of the flow cell is 1 cm, while the microfluidic system has an optical path of 0.01 cm. In addition, when the enzyme is immobilized at a point, and the substrate passes through that point, enzyme-substrate interactions are favored, making it possible to reach maximum speed with lower substrate concentrations. This difference is manifested in the variation of intensity obtained with both methods, achieving a higher signal intensity with the microfluidic system.

**References**

1. Écija-Arenas Á, Román-Pizarro V, Fernández-Romero JM (2021) Usefulness of Hybrid Magnetoliposomes for Aminoglycoside Antibiotic Residues Determination in Food Using an Integrated Microfluidic System with Fluorometric Detection. J Agric Food Chem 69:6888–6896. https://doi.org/10.1021/acs.jafc.1c01571

2. Saiyed ZM, Sharma S, Godawat R, et al. (2007) Activity and stability of alkaline phosphatase (ALP) immobilized onto magnetic nanoparticles (Fe3O4). J Biotechnol 131:240–244. https://doi.org/10.1016/J.JBIOTEC.2007.06.017

3. Écija-Arenas Á, Román-Pizarro V, Fernández-Romero JM, Gómez-Hens A (2016) Separation and purification of hydrophobic magnetite-gold hybrid nanoparticles by multiphase density gradient centrifugation. Microchimica Acta 183:2005–2012. https://doi.org/10.1007/s00604-016-1838-z

4. Román-Pizarro V, Ramírez-Gutiérrez M, Gómez-Hens A, Fernández-Romero JM (2020) Usefulness of magnetically-controlled MNPs-enzymes microreactors for the fluorimetric determination of total cholesterol in serum. Talanta 208:120426. https://doi.org/10.1016/j.talanta.2019.120426
